# Comparing Brief Internet-Based Compassionate Mind Training and Cognitive Behavioral Therapy for Perinatal Women: Study Protocol for a Randomized Controlled Trial

**DOI:** 10.2196/resprot.5332

**Published:** 2016-04-15

**Authors:** Alex R Kelman, Meagan L Stanley, Alinne Z Barrera, Michelle Cree, Yotam Heineberg, Paul Gilbert

**Affiliations:** ^1^ Palo Alto University Palo Alto, CA United States; ^2^ i4health Palo Alto, CA, CA United States; ^3^ Derbyshire Healthcare NHS Foundation Trust Derby United Kingdom; ^4^ Compassionate Mind Foundation Derby United Kingdom; ^5^ Stanford University, Center for Compassion and Altruism Research and Education Palo Alto, CA United States

**Keywords:** perinatal depression, comparative trial, Internet intervention, Amazon Mechanical Turk

## Abstract

**Background:**

Depression that occurs during the perinatal period has substantial costs for both the mother and her baby. Since in-person care often falls short of meeting the global need of perinatal women, Internet interventions may function as an alternate to help women who currently lack adequate access to face-to-face psychological resources. However, at present there are insufficient empirically supported Internet-based resources for perinatal women.

**Objective:**

The aim of this study is to compare the relative efficacy of Internet-based cognitive behavioral therapy (CBT) to a novel Internet-based compassionate mind training approach (CMT) across measures of affect, self-reassurance, self-criticizing, self-attacking, self-compassion, depression, and anxiety. While CBT has been tested and has some support as an Internet tool for perinatal women, this is the first trial to look at CMT for perinatal women over the Internet.

**Methods:**

Participants were recruited through Amazon Mechanical Turk (MTurk) and professional networks. Following completion of demographic items, participants were randomly assigned to either the CBT or CMT condition. Each condition consisted of 45-minute interactive didactic and follow-up exercises to be completed over the course of two weeks.

**Results:**

Post course data was gathered at two weeks. A 2x2 repeated measures analysis of variance will be conducted to analyze differences between conditions at post course.

**Conclusions:**

The implications of the trial will be discussed as well as the strengths and limitations of MTurk as a tool for recruitment. We will also briefly introduce the future directions along this same line of research.

**Trial Registration:**

ClinicalTrials.gov NCT02469324; https://clinicaltrials.gov/ct2/show/NCT02469324 (Archived by WebCite at http://www.webcitation.org/6fkSG3yuW)

## Introduction

Mood changes during and after pregnancy are common although they do not always result in clinically significant depressive episodes. However, both severe and the moderate/milder forms of depression during the perinatal period and postnatal can be a source of major problems to mothers, their babies, and even other family members. Symptoms of postpartum depression (PPD) include, but are not limited to, concentration difficulties, poor sleep, heightened anxiety, feelings of inadequacy, ruminative fears and compulsive thoughts, loss of attachment sentiments, and guilt feelings [1]. The physiological effects of prenatal depression such as elevated cortisol can adversely affect the baby’s development and result in negative consequences that remain undetected into adulthood [2].

The costs of PPD, both for the mother and her child, are concerning, especially since the incidence of PPD reaches 7.1% in the United States (US) and is as high as 19.2% when minor depressive episodes are included [3]. It is estimated that the global incidence of PPD is even higher than within the United States [4], suggesting a pressing global health concern for women and their children. Additionally, there is research that demonstrates a relationship between lower income areas and higher PPD prevalence [5], demonstrating that communities who already lack access to adequate health and mental health resources are further affected by untreated PPD in their communities.

Even though the human and financial costs of untreated PPD are well-established, adequate resources to address the global need of women suffering from PPD or who are at risk of developing PPD do not yet exist. Presently, the first line of psychological treatment for PPD is cognitive behavioral therapy (CBT). However, in a review of five randomized controlled trials (RCTs) comparing CBT with standard postpartum care, only two trials demonstrated better outcomes in the CBT group [6]. While preliminary Interpersonal Therapy (IPT), with a focus on handling the interpersonal disputes that occur during childbirth and childcare, has shown some promise in addressing this pressing need [7]. The first line of treatment for PPD continues to be medication [8], particularly selective serotonin reuptake inhibitors (SSRIs), which show some level of efficacy and perform equal to psychotherapy in randomized trials [8,9]. However, there are concerns regarding the safety of antidepressants for pregnant and nursing women [10].

There are many practical limitations to treatment seeking, not least being the availability of appropriately qualified therapists. In addition, issues such as stigma, childcare difficulties, lack of knowledge, and financial constraints can inhibit help seeking [11]. As such, psychological resources that do not require women to leave their homes may allow for more women to participate in psychological interventions. In general, Internet interventions can be private and used anonymously, accessed repeatedly at any time, and from any location, and Internet interventions may provide a much-needed service to users who feel marginalized or stigmatized [12]. Clatworthy’s [13] meta-analysis did not find a relationship between the length of an in-person preventative intervention and its effectiveness, suggesting that increasing the length of psychological interventions is not an important factor in enhancing benefit. However, there is research to show that interventions based on psychological models tend to be more effective than interventions that are not based on these models [13], meaning that researchers seeking to create new resources for women should maintain fidelity to empirically supported psychological models.

As a result of the limitations that preclude many women from seeking in-person resources, Internet interventions offer promise in providing care with less financial and practical barriers. Additionally, Internet interventions can be used as adjuncts to in-person treatments or replacements in other cases [14], and perhaps offer even more promise for reducing health disparities. Nonconsumable interventions, or those that can be reused with minimal cost for each additional participant, can help to close the gap in services through offering assistance without needing the support of a clinician or operator on the other end [15]. Currently, there is a dearth of research on Internet interventions for perinatal women or to address PPD [16].

Importantly, over the last 20 years different psychotherapies have been developed that are increasingly rooted in the scientific understanding of psychological processes, including those associated with attachment. For example, compassion focused therapy (CFT) is based on evolutionary insights of brain function, particularly the importance of building and developing affiliative and prosocial relationships with self and other [17,18]. This model in particular articulates the role of affiliative emotion and motivation in threat regulation and how these can be cultivated through (1) addressing issues of self-criticism and shame and (2) with affiliation building exercises (compassion cultivation). This is important because at the heart of many depression difficulties is a harshly self-critical relationship with the self and at times nonaffiliative relations with self and with others. In addition, helping to build affiliative relationships between mothers and their babies is obviously important. There is now considerable evidence that compassion cultivation has an impact on a range of physiological systems including the immune system, frontal cortex, and cardiovascular systems [19]. It also promotes prosocial and empathic behavior [20]. This makes compassion cultivation an especially important focus for intervention for this population.

The present study therefore seeks to assess the efficacy of an Internet-based Compassionate Mind Training (CMT) program, the intervention component of CFT, compared to a standard CBT program in a sample of childbearing and perinatal women. As there is currently a lack of Internet interventions for maternal mental health, the research team intends to assess the relative efficacy of CMT as a resource for women during this period.

It is expected that participants randomized to both Internet-based CMT and CBT will demonstrate improved affect as well as reduced depression and anxiety at similar, if not better, levels which are the secondary outcome measures of this trial. However, in terms of the primary outcome measures, we expect that Internet-based CMT will provide unique benefit above and beyond CBT in the constructs of self-reassurance, self-criticizing, self-attacking, and self-compassion, as CMT is specifically targeted towards these constructs.

## Methods

### Participants and Procedures

Participants were recruited through two primary sources: online through Amazon Mechanical Turk (MTurk) and through professional networks. Recruitment for the present study occurred between April 2015 to September 2015.

Upon navigating to the landing page, participants were asked to review and agree to informed consent (IC) before beginning the study. For a copy of the IC for the present study, see Multimedia Appendix 1. Participants who met criteria for inclusion were then randomized to either the CBT (n=60) or CMT (n=60) condition. As the trial was designed to assess the efficacy among perinatal women of an Internet-based CMT relative to an Internet-based CBT intervention, a control group was not employed. To ensure equivalent numbers of participants were randomized to both conditions, an embedded algorithm was used.

Each condition was divided into equal parts (a) and (b) with condition-specific content reflective of the theoretical orientation of the intervention. In part (a), participants completed a didactic, which included written materials and brief experiential exercises. The didactic portion of the course provided an introduction to the approach and was created to take approximately 45 minutes to complete. Following completion of part (a), participants received an automatically generated email based on their assigned condition. The email included follow-up exercises for the CBT condition and audio meditations for the CMT condition. At this point, participants were informed that they would receive additional emails at Day 4, Day 7, and Day 14 of the course.

Based on a power analysis to detect an effect of .4 at alpha .05 with 80% power, this study needed to include a minimum of 23 participants for each condition who complete the post course assessment at two weeks. The standardized effect size was estimated using [21] and the meaning of this size is .25 for small, .5 for medium and 1.00 for large. The designated medium effect size is consistent with what is universally agreed upon in published mindfulness research [22]. However, the research team tried to recruit 60 participants for each condition in order to account for attrition that is inherent in clinical trial, especially ones conducted over the Internet.

### Inclusion Criteria

In order to be included in the present study, participants needed to be proficient in English, over the age of 18 years, currently pregnant, pregnant within the last year, or intending to become pregnant in the future. Interested women who met eligibility criteria were included in the present study regardless of depression status at study entry.

### Exclusion Criteria

Participants were excluded from participation in the present study if they were male, under the age of 18 years, not intending to become pregnant in the future, not pregnant, and not pregnant within the last year.

### Treatment Conditions

For the present trial, participants were randomly assigned to either the Internet-based CBT or CMT condition. The CBT condition, which did not have pregnancy-specific information within the course, included lessons on (1) thoughts, (2) activities, (3) assertiveness, and (4) sleep. Ricardo Muñoz and the Institute for International Internet Interventions (i4health) team developed the materials for this condition, which is based on standard CBT constructs [23]. The CMT condition, which had a limited amount of pregnancy-specific content only in module three, included (1) finding ourselves here in the flow of life, (2) old brain, new brain, (3) the three circles of affect regulation and pregnancy, and (4) cultivating the compassionate self.

### Outcome Measures

Self-criticizing, self-attacking, and self-reassurance were assessed through the Forms of Self-Criticizing/Attacking and Self-Reassurance Scale (FSCRS; [24]). This measure identifies levels of self-criticizing and attacking and capacity for one to engage in self-reassurance [24]. In a sample of female college students, Cronbach alpha for inadequate self was at .90 and .86, for hated self and reassured self-subscales, respectively [24]. The FSCRS was administered to participants before and after the didactic portion of the course.

A Likert-scale affect item (“how would you rate your mood currently?” from 0-very bad to 7-very good) was employed to assess current affective state of participants at two time points during the course. The affect item was presented to participants at baseline and following the didactic portion of the course.

Depression and anxiety were assessed through the Patient Health Questionnaire-4 (PHQ-4; [25]. The PHQ-4 is a self-report measure that has two items to assess depression through the PHQ-2 and two items to assess anxiety through the Generalized Anxiety Disorder scale-2 (GAD-2) [26]. Based on a sample from the general population, Cronbach alpha was .78 for PHQ-2 and .75 for GAD-2 [25]. When compared to the Structured Clinical Interview for DSM-IV Disorders (SCID-4), a scaled score of 3 on the PHQ-2 scale demonstrated sensitivity of 87% and specificity of 78% for Major Depressive Disorder (MDD), and it demonstrated comparable diagnostic performance relative to longer measures [27]. The PHQ-4 was administered before the didactic portion of the course and following completion of post course measures at two weeks.

Self-compassion shifts were measured through the short form of the Self-Compassion Scale (SCS-SF; [28]). The SCS-SF measures how individuals respond to themselves during times of stress in order to assess level of self-compassion [28]. The SCS-SF has a near perfect correlation with the longer version of the measure at .97. It has a Cronbach alpha of .86 in the general population in the United States [28]. The SCS-SF was given to participants before the didactic portion of the course and after completion of post course measures at two weeks*.*

Participant feedback items were asked at post course to elicit information on use (ie, the number of times materials were practiced during each week, overall perceived helpfulness of the materials). While no formalized measure was given to elicit feedback, the information on perceived helpfulness and utilization was gathered in order to provide participant-directed guidance for updating the CMT condition materials.

**Figure 1:**
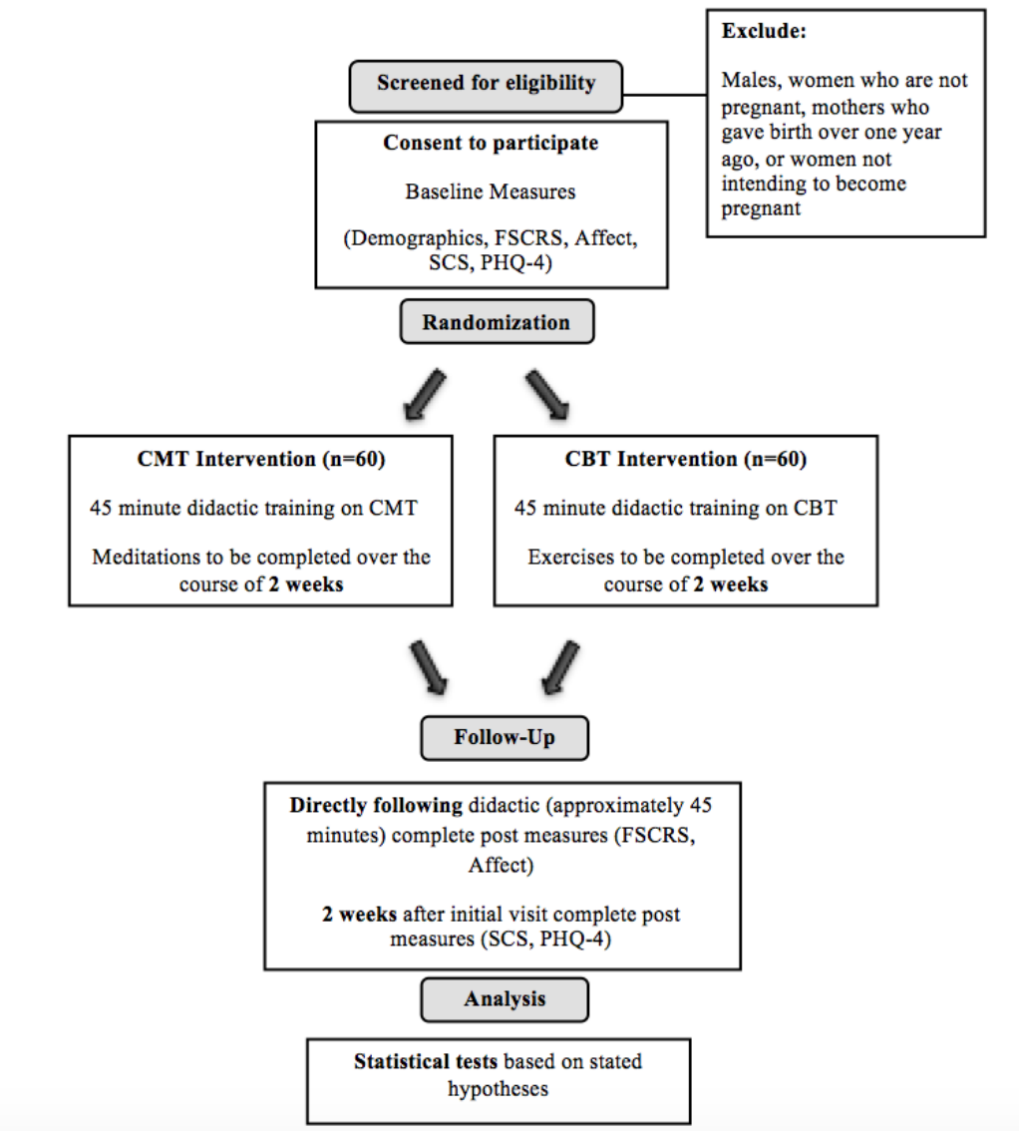
Design of the study.

For a visual representation of the study design, refer to Figure 1.

### Ethics

The present study received approval by the Palo Alto University Institutional Review Board and is being conducted under the supervision of Alinne Barrera, PhD.

### Analysis

The present study seeks to compare the efficacy of two theoretical approaches using a randomized conditional approach with outcome comparisons at baseline, throughout the course period, and at the post course two weeks following randomization. Pre course equivalence between participants in each condition will follow analytical procedures recommended by the CONSORT 2010 Statement [29]. A 2x2 mixed analysis of variance (ANOVA) will be run for each measure at baseline and follow-up. The between subjects factor in the ANOVA will be condition and the within subjects factor will be the outcome measure. A main effect of time and a main effect of condition will be analyzed to determine if there is a difference between CBT and CMT on each of the outcome measures.

## Results

The present study has the aim to assess relative efficacy of Internet-based CMT compared to CBT for perinatal women through conducting a randomized controlled trial. While there is currently some research to support Internet-based CBT for perinatal women [30-32], there is still a clear need for additional Internet-based psychological resources for this vulnerable population. As such, research into additional Internet-based psychological programs for perinatal women is important to meet this need.

*Trial status:* as of April 2015, participants were being enrolled in the study. Participants were recruited continuously throughout the enrollment period. Recruitment ended in September 2015.

## Discussion

The current study is a two condition randomized controlled trial comparing brief Internet-based CBT and CMT in enhancing the well-being of perinatal women and women with future intentions of pregnancy. As far as the research team is aware, this is the first trial to assess the relative efficacy of an automated compassion meditation program to a CBT program for working with current and future perinatal women over the Internet. The results are expected to impact the future direction of Internet interventions for perinatal women. It is important to note that the trial is not recruiting women based on depression status, but rather a sample of general population perinatal women. As such, the research team will be unable to make decisive conclusions about the treatment effectiveness of the CMT course based on results from this trial. However, upon completion of this preliminary study, future studies will employ a prevention model approach in order to assess the preventative effectiveness of CMT with respect to PPD development among women in their second and third trimesters. Participant feedback from the current study will allow the research team to update the CMT course material in order to better meet the needs of the target population in phase two of the trial. Finally, and with the intention to make the resources widely available to a global population of women, the research team hopes to translate the study materials into additional languages in order to better serve a more diverse group of perinatal women.

The more that is known about differential effects of Internet interventions, the more researchers can start to tailor certain Internet interventions towards the population that is likely to show benefit from that particular content. To this end, in addition to examining the primary and secondary outcomes of this current pilot study, the research team aims to explore several additional questions. For example, perhaps some groups within the included sample of this study will benefit more from utilizing one condition relative to the other. Alternatively, perhaps the data may indicate that women from certain parts of the world respond better to one condition of the intervention relative to the other. Finally, perhaps the effectiveness of each condition of the intervention will be moderated by the levels of depression the woman are currently experiencing at study entry or the demographics of those women. These are all possible avenues for exploration that the research team will aim to examine once all data have been collected for this pilot trial. The long-term goal for this line of research will be to contribute to the psychological community’s understanding of how best to create and disseminate Internet interventions that are targeted towards women who may benefit from them the most.

In addition to the important implications that exist, there are also several limitations to the current study. First, since the majority of recruitment is being conducted using the MTurk system, we lack the capacity to verify the demographics of the individuals who take part in the study. While this is a known issue for MTurk [33], there is evidence to support that running research with an MTurk sample tends to produce near equivalent results as trials conducted in a laboratory setting [34]. Additionally, MTurk workers tend to be more demographically diverse than standard Internet samples and college students that comprise samples of many trials [34], which is a strength to using MTurk as a recruitment tool.

MTurk workers received compensation when they completed post course data at two weeks. There is an inherent limitation with a highly motivated sample of women who will receive compensation for completing post course data in order to receive payment for their participation. Internet-based research has a high level of attrition [35], and thus the completion rates witnessed with an MTurk sample are unlikely to be generalizable to a broader group of international perinatal women. As such, it is impossible to know what actual attrition rates would be in a non-MTurk group of women if the majority of recruitments comes from this source.

Perhaps the most important and glaring limitation of the current trial is the lack of a more distal time point for measurement of outcome variables. The limited follow-up with participants, only occurring at two weeks post course, does not allow the team to make inferences about the longer-term maintenance of improvements in either condition. As the ultimate goal of psychological Internet interventions is to decrease suffering on a global scale, knowledge of longer-term utility of these resources is crucial. In order to account for this limitation during future research along this same line, we will add another follow-up after pregnancy, which can be up to six months after providing baseline data.

## Appendix

Multimedia Appendix 1 Informed Consent document for the study.

## Abbreviations

ANOVA: analysis of variance
CBT: cognitive behavioral therapy
CFT: compassion focused therapy
CMT: compassionate mind training
FSCRS: Forms of Self-Criticizing/Attacking and Self-Reassurance Scale
IC: informed consent
IPT: interpersonal psychotherapy
MDD: major depressive disorder
MTurk: Amazon Mechanical Turk
PHQ-4: Patient Health Questionnaire-4
PPD: postpartum depression
RCT: randomized controlled trial
SCS-SF: Self-Compassion Scale-Short Form
SSRI: selective serotonin reuptake inhibitor
