# Supplementary material for: Comparing Brief Internet-Based Compassionate Mind Training and Cognitive Behavioral Therapy for Perinatal Women: Study Protocol for a Randomized Controlled Trial
Source: JMIR Res Protoc. 2016 Apr 15;5(2):e65. doi: 10.2196/resprot.5332 (PMC4851724; doi:10.2196/resprot.5332)
Supplement: Multimedia Appendix 1 [file resprot_v5i2e65_app1.pdf]

A. PURPOSE AND BACKGROUND

The current study is a dissertation conducted by Alex Kelman, M.S. under the supervision of Alinne Barrera, Ph.D., both from Palo Alto University. The researchers are conducting a study to learn about the therapeutic benefit of a brief Internet intervention for improving well-being.

You are being asked to participate in this study because you are currently pregnant, intend to become pregnant in the future, or have given birth within the last year.

B. PROCEDURES

If you agree to be in this study, the following will happen.

You will complete a set of questions that will help us gain background information about you. You will then complete brief questionnaires to give us a sense of your well-being before the study. You will complete a 45-minute brief didactic course, where you will learn techniques to enhance well-being. You will then complete some of the questionnaires again, in order for us to see if the didactic course helped. You will then receive an email with follow-up exercises for you to complete, at your pace, over the next 2 weeks. Finally, you will receive an email that will ask you to fill out the remainder of the questionnaires you completed at step 1. This will allow us to see if the whole course was helpful.

C. RISKS/DISCOMFORTS

It is not uncommon for participants in research studies to experience some amount of anxiety. You can choose not to participate in the study or withdraw at any point if you do not feel that continuing is in your best interest.

1. Confidentiality: Participation in research may involve a loss of privacy, but information about you will be handled as confidentially as possible. Keep in mind that email addresses will be collected during this study, which means that your information will not be entirely confidential as a result. The risk of this lack of de-identification is that your answers to the course material will be tied to your email address. However, special precautions will be taken to protect the identities of participants in the study and the confidentiality of all information provided. When the data is analyzed, all records will be de-identified so information we gather about you will not be tied to identifying information (e.g. email address, name). Additionally, no individual identities will be used in any reports or publications resulting from this study. In the event that a participant discloses immediate plans to harm herself or someone else, the researchers will notify the participant they are obligated to make a report and will then reach out to the appropriate agency regarding the risk issue.

D. BENEFITS

Since the Internet intervention is based on evidence-based treatments, it is possible that participants will receive some benefit from participating; however, you can withdraw from the study at any point if you do not feel that you are benefiting from the course. Additionally, you will learn skills that should continue to help after conclusion of the study and resources from the study will be available to you after the study is over.

E. ALTERNATIVES

There are no alternative procedures for this study.

F. PAYMENT

You will not be paid for participating in this study. However, you will be able to keep and reuse the information that you learned during the course of the study.

G. QUESTIONS

The investigator, Alex Kelman, has explained this study to you. If you have any other questions about the study, you may email the investigator at akelman@paloaltou.edu.

Questions about your rights as a participant in this study may be presented to the IRB Chair by mail at Palo Alto University, IRB, 1791 Arastradero Road, Palo Alto, CA 94304 or by phone at 650-433-3827.

You may print a copy of this consent form at this time for your records.
